# Associations of P Score With Real-World Survival Improvement Offered by Adjuvant Chemotherapy in Stage II Colon Cancer: A Large Population-Based Longitudinal Cohort Study

**DOI:** 10.3389/fonc.2021.574772

**Published:** 2021-02-24

**Authors:** Qi Liu, Zezhi Shan, Dakui Luo, Sheng Zhang, Qingguo Li, Xinxiang Li

**Affiliations:** ^1^ Department of Colorectal Surgery, Fudan University Shanghai Cancer Center, Shanghai, China; ^2^ Department of Oncology, Shanghai Medical College, Fudan University, Shanghai, China

**Keywords:** prognostic scoring system, stage II, colon cancer, adjuvant chemotherapy, SEER

## Abstract

**Background:**

Based on a prognostic scoring system (*P* score) proposed by us recently, this retrospective large population-based and propensity score-matched (PSM) study focused on predicting the survival benefit of adjuvant CT in stage II disease.

**Methods:**

Patients diagnosed with stage II colon cancer (N = 73397) were identified from the Surveillance, Epidemiology, and End Results database between January 1, 1988 and December 31, 2005 and divided into the CT and non-CT groups. PSM balanced the patient characteristics between the CT and non-CT groups.

**Results:**

The magnitude of CSS improvement among patients treated with adjuvant CT was significantly associated with the P score, score 8 [hazard ratio (HR) = 0.580, 95% confidence interval (CI) = 0.323–1.040, P = 0.067] was associated with a much higher increased CSS benefit among patients treated with adjuvant CT as compared to score 2* (*, including scores 0, 1, and 2; HR = 1.338, 95% CI = 1.089–1.644, P = 0.006).

**Conclusions:**

High *P* scores were demonstrated to be associated with superior survival benefit of adjuvant CT. Therapy decisions of adjuvant CT in stage II colon cancer could be tailored on the basis of tumor biology, patient characteristics and the *P* score.

## Background

Colon cancer was the third most commonly diagnosed malignant tumor worldwide (1). Despite that adjuvant chemotherapy (CT) was widely applied clinically with clearly established evidence of survival benefit for stage III colon cancer, its efficacy for stage II colon cancer was yet controversial (2–5). The famous Quick, Simple, and Reliable (QUASAR) prospective trial reported a pool survival benefit for patients with stage I–III colorectal cancer after CT as compared to surgery alone; however, it failed to demonstrate the efficacy of CT among stage II colon cancer subgroup (3).

Although direct evidence of benefit was lacking, the American Society of Clinical Oncology (ASCO) clinical guidelines recommended adjuvant CT for high-risk stage II colon cancer (including patients with inadequately sampled nodes, T4 lesions, perforation, or poorly differentiated histology) (6). Also, the European Society for Medical Oncology (ESMO) proposed similar recommendations (7). However, the efficacy of adjuvant CT in stage II colon cancer with high-risk factors was still controversial (8). Two retrospective clinical studies reported the survival benefit of adjuvant CT in stage II colon cancer with high-risk factors (9, 10). But more clinical studies suspected the survival benefit of adjuvant CT in the so-called high-risk stage II colon cancer (8, 11–14).

A wide clinical application of adjuvant CT in high-risk stage II colon cancer in spite of the uncertainty of survival benefit makes the studies of adjuvant CT in stage II colon cancer quite necessary. Thus, the purpose of the study was to predict the survival effect among stage II colon cancer with the prognostic scoring system proposed in our previous study (15) in order to obtain an improved prognostic prediction of stage II colon cancer with different *P* scores after receiving adjuvant CT.

## Methods

### Study Design and Data Source

In this study, patients were recruited from the Surveillance, Epidemiology, and End Results (SEER) Program of the United States National Cancer Institute, released in 2018. The SEER database was an authoritative and public source of information on cancer incidence, mortality, prevalence, lifetime risk statistics, and survival in the United States. We used SEER-Stat software (version 8.3.5) to get access in this study.

**Figure 1:**
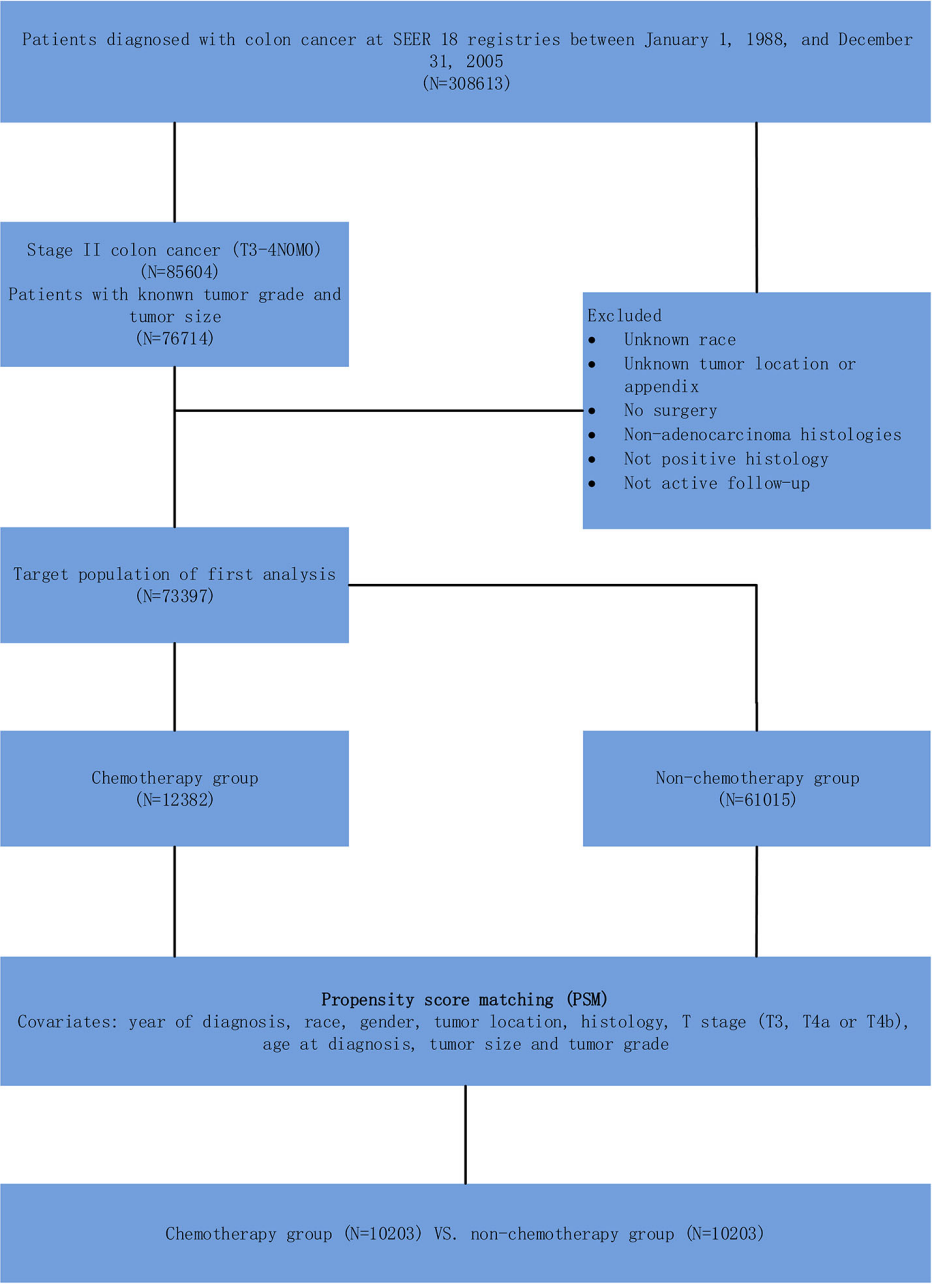
Schematic representation of patient population selected from SEER database.

As shown in **Figure 1**, we identified 73,397 stage II colon cancer patients from January 1, 1988 to December 31, 2005 for the initial analysis. Next, patients diagnosed within these years were included in our study because the SEER database started recording detailed tumor size from 1988 (tumor size was essential for the prognostic scoring system) and we wanted to allow for 10 years of follow-up (the follow-up of the present study ended in 2015). We excluded patients with unknown information of some significant prognostic factors, such as tumor grade, tumor size, race, tumor location (appendix was not included from this study), and so on. Also, patients without surgery or adenocarcinoma histology or positive histology or active follow-up were excluded from our target population.

### Prognostic Scoring System

**Figure 2:**
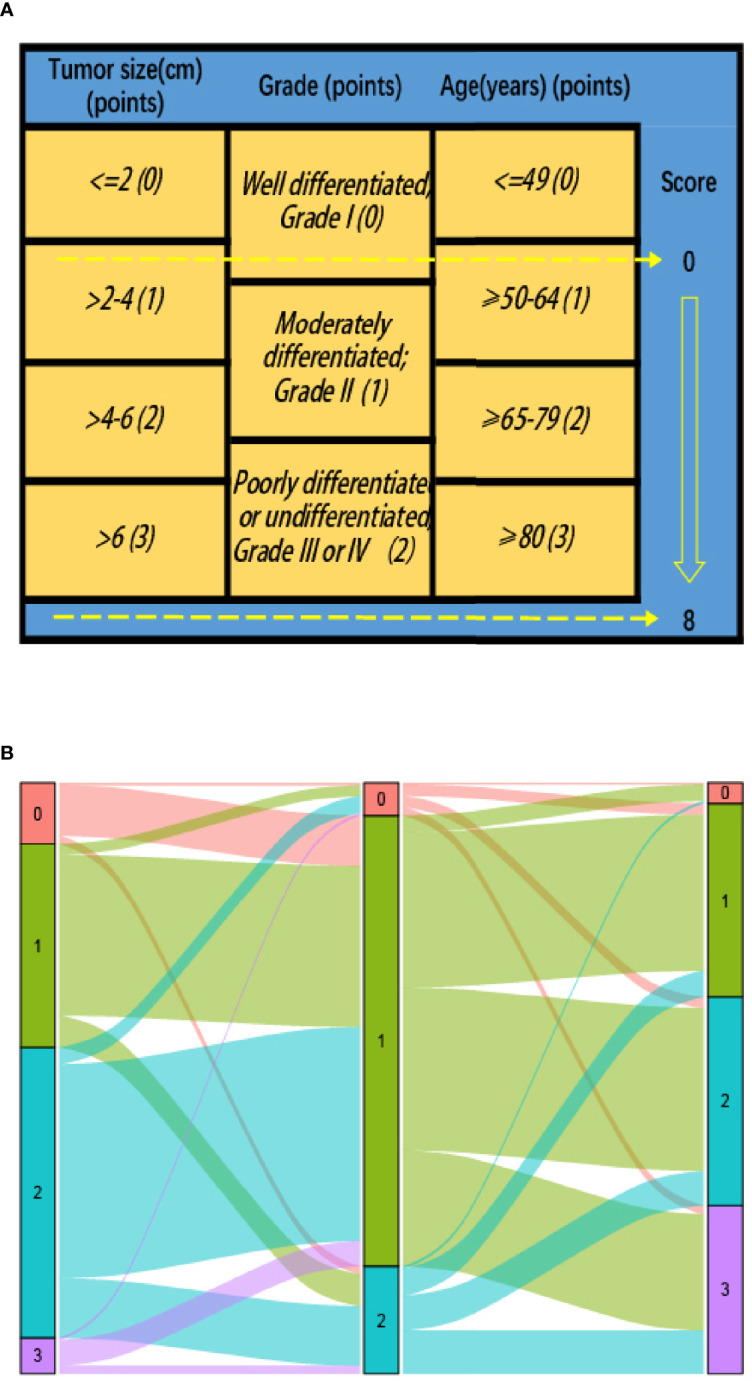
**(A)** Modified prognostic scoring system (*P* score) in stage II colon cancer patients: risk-stratifications; **(B)** Graphical summary of tumor size, tumor grade and age, and their subgroup distribution.

To investigate the benefit of adjuvant CT after surgery, we used the newly proposed prognostic scoring system (*P* score) and the detailed scoring rules were showed in our previous study (15). Since only 457 patients (0.6%) were diagnosed with undifferentiated tumor grade (grade IV), grade III and grade IV were merged. As shown as **Figure 2**, *P* score (that is the prognostic scoring system) that was obtained based on the tumor size, tumor grade, and age at diagnosis ranged from 0–8 with a score of 0 indicating the best prognosis and those with a score of 8 indicating the poorest survival.

### Statistical Analyses

In this study, different clinicopathologic factors were compared between the CT and non-CT groups using Pearson’s chi-squared test for categorical variables. The primary endpoint used for comparison were cause-specific survival (CSS). We also constructed some multivariate Cox proportional hazard models to evaluate the survival benefit of adjuvant CT.

As an observational study, significant bias might be introduced by inherent differences between patients receiving or not receiving adjuvant CT. In addition, we defined the predicted probability of treatment as a propensity score to balance the clinicopathologic factors between the CT and non-CT groups in SEER cohort using the following baseline characteristics that strongly related to the survival but less strongly related to the treatment: year of diagnosis, race, gender, tumor location, histology, T stage (including T3, T4a or T4b), age at diagnosis, tumor size, and tumor grade (16). Patients receiving adjuvant CT were matched on a one-to-one basis with patients without receiving adjuvant CT (**Figure 1**). We performed the matching based on the nearest-neighbor methods. The propensity score indicated the probability of the patients receiving the adjuvant CT based on the baseline characteristics. In our study, we preformed the statistical analysis mainly using SPSS version 22 (IBM Corporation, Armonk, NY, USA), and two-sided *P* value < 0.05 was considered statistically significant.

## Results

### Patient Characteristics

The median follow-up time of the censored patients in the SEER cohort was 9.67 years, following which, at the end of the follow-up time, 13,880 (18.9%) patients died because of colon cancer. Of the initial cohort, 61,015 patients (83.1%) were stratified into the non-CT group, and 12,382 patients (16.9%) were stratified into the CT group. **Table 1** summarized the patients’ baseline demographic characteristics. All demographic characteristics were statistically related to the receipt of the adjuvant CT (P < 0.001). The patients diagnosed during later years, male patients, T4 stage, younger patients, patients with large tumor size, and patients with high tumor grade were more likely to receive adjuvant CT (P < 0.001).

**Table 1:** Baseline characteristics of the overall cohort by the receipt of adjuvant CT before PSM.

Characteristic	No. of Patients (%)		*P*
	CT Group (n = 12,382)	Non-CT Group (n = 61,015)	
Year of diagnosis			<0.001
1988–1992	1,282 (10.4)	10,735 (17.6)	
1993–1997	2,747 (22.2)	13,222 (21.7)	
1998–2001	3,791 (30.6)	16,143 (26.5)	
2002–2005	4,562 (36.8)	20,915 (34.3)	
Race			<0.001
White	10,237 (82.7)	51,884 (85.0)	
Black	1,121 (9.1)	5,404 (8.9)	
Other	1,024 (8.3)	3,727 (6.1)	
Gender			<0.001
Male	6,221 (50.2)	28,162 (46.2)	
Female	6,161 (49.8)	32,853 (53.8)	
Tumor location			<0.001
Cecum	2,728 (22.0)	15,141 (24.8)	
Ascending colon	2,085 (16.8)	12,174 (20.0)	
Hepatic flexure	782 (6.3)	4,393 (7.2)	
Transverse colon	1,391 (11.2)	7,074 (11.6)	
Splenic flexure	604 (4.9)	2,931 (4.8)	
Descending colon	935 (7.6)	3,983 (6.5)	
Sigmoid Colon	3,857 (31.2)	15,319 (25.1)	
Histology			<0.001
Adenocarcinoma	11,017 (89.0)	55,221 (90.5)	
Mucinous adenocarcinoma	1,279 (10.3)	5,474 (9.0)	
Signet ring cell carcinoma	86 (0.7)	320 (0.5)	
T stage			<0.001
T3	9,671 (78.1)	52,364 (85.8)	
T4a	1,253 (10.1)	5,817 (9.5)	
T4b	1,458 (11.8)	2,834 (4.6)	
Age at diagnosis (years)			<0.001
≤49	1,900 (15.3)	2,377 (3.9)	
>49–64	4,377 (35.3)	9,361 (15.3)	
>64–79	5,439 (43.9)	27,704 (45.4)	
>79	666 (5.4)	21,573 (35.4)	
Tumor size (cm)			<0.001
≤2	494 (4.0)	3,202 (5.2)	
>2–4	3,801 (30.7)	22,152 (36.3)	
>4–6	4,230 (34.2)	21,096 (34.6)	
>6	3,857 (31.2)	14,565 (23.9)	
Tumor grade			<0.001
Grade I	854 (6.9)	5,338 (8.8)	
Grade II	8,950 (72.3)	44,944 (73.7)	
Grade III/IV	2,578 (20.8)	10,732 (17.6)	

CT, chemotherapy; PSM, propensity score matching.

### Survival Benefit of Adjuvant Chemotherapy According to *P* score Before Propensity Score Matching

Considering that the scores 0 and 1 accounted for only <0.1 and 0.4% of the overall cohort, respectively, the scores 0, 1, and 2 were then classified as the same score. As shown in **Figure S1** after multivariate Cox and Kaplan–Meier analyses of CSS, the magnitude of CSS improvement among patients treated with adjuvant CT was significantly associated with the *P* score, score 8 [hazard ratio (HR) = 0.580, 95% confidence interval (CI) = 0.323–1.040, P = 0.067] was associated with a much higher increased CSS benefit among patients treated with adjuvant CT compared to score 2^*^ (*, including scores 0, 1, and 2; HR = 1.338, 95% CI = 1.089–1.644, P = 0.006). In other words, the decrease of 10-year CSS rates among the non-CT group with the increase of *P* score was much faster than the CT group [the decrease of CSS with the increase of *P* score in colon cancer has been demonstrated in our previous study (15)]. In the CT group, the 10-year CSS rate decreased gradually as the score increased only with the exception that the 10-year CSS was higher in score 8 (78.7%) than that in score 7 (74.9%), and we thought it was plausible to conclude it was mainly due to the substantial survival benefit of adjuvant CT in score 8.

### Survival Benefit of Adjuvant Chemotherapy According to *P* score After Propensity Score Matching

**Figure 3:**
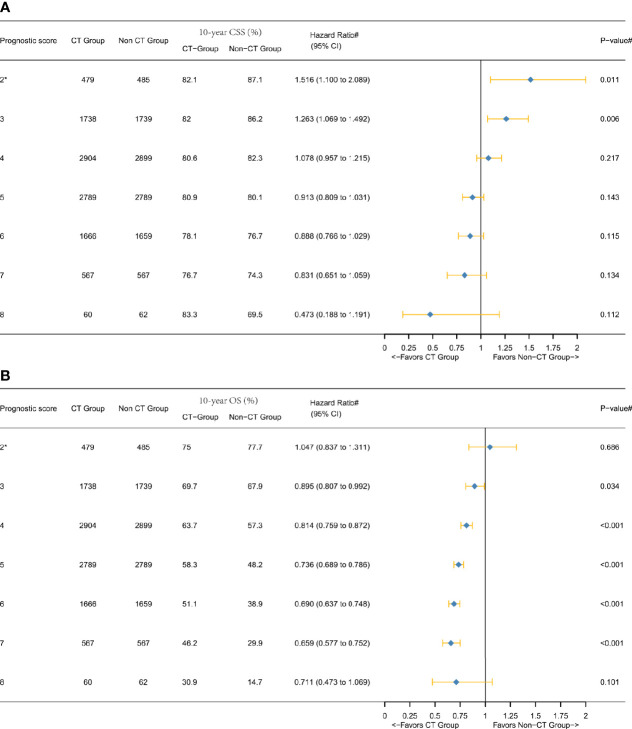
Hazard ratios comparing the survival between CT and non-CT groups according to the *P* score in the overall cohort after PSM comparing **(A)** CSS and **(B)** overall survival (OS). (2*) Including *P* scores 0, 1, and 2. (#) Multivariate analysis adjusted by the year of diagnosis, race, gender, tumor location, histology, T stage (including T3, T4a, or T4b), age at diagnosis, tumor size, and tumor grade.

As shown in **Table 2**, PSM generated 10,203 patients in the CT group and 10,203 patients in the non-CT group. The median follow-up time among the censored patients was 11.83 years. At the end of the follow-up time, 3,844 (18.8%) patients died of colon cancer. As shown in **Figure 3A**, multivariate Cox and Kaplan–Meier analyses of CSS found that the magnitude of CSS improvement among patients treated with adjuvant CT was also significantly associated with the *P* score and the HRs between CT and non-CT groups decreased gradually when the score increased without exception. Score 8 (HR = 0.473, 95% CI = 0.188–1.191, P = 0.112) was associated with a much higher increased CSS benefit among patient with adjuvant CT as compared to that of score 2^*^ (*, including scores 0, 1, and 2; HR = 1.516, 95% CI = 1.100–2.089, P = 0.011), and the phenomenon was more obvious than in the overall cohort before PSM. The decrease of 10-year CSS rate among the non-CT group with the increase of *P* score was much faster than that among the CT group [the decrease of CSS with the increase of *P* score in colon cancer has been demonstrated in our previous study (15)]; the 10-year CSS rate was even higher in score 8 (83.3%) than score 7 (76.7%) among the CT group, and we thought it was plausible to conclude it was mainly due to the substantial survival benefit of adjuvant CT in score 8.

**Table 2:** Baseline characteristics of the overall cohort by the receipt of adjuvant CT after PSM.

Characteristic	No. of Patients (%)		*P*
	CT Group (n = 10,203)	Non-CT Group (n = 10,203)	
Year of diagnosis			0.943
1988–1992	1,063 (10.4)	1,083 (10.6)	
1993–1997	2,271 (22.3)	2,290 (22.4)	
1998–2001	3,115 (30.5)	3,091 (30.3)	
2002–2005	3,754 (36.8)	3,739 (36.6)	
Race			0.958
White	8,832 (86.6)	8,821 (86.5)	
Black	769 (7.5)	780 (7.6)	
Other	602 (5.9)	602 (5.9)	
Gender			0.966
Male	5,155 (50.5)	5,158 (50.6)	
Female	5,048 (49.5)	5,045 (49.4)	
Tumor location			1.000
Cecum	2,332 (22.9)	2,335 (22.9)	
Ascending colon	1,741 (17.1)	1,733 (17.0)	
Hepatic flexure	581 (5.7)	584 (5.7)	
Transverse colon	1,096 (10.7)	1,088 (10.7)	
Splenic flexure	418 (4.1)	424 (4.2)	
Descending colon	695 (6.8)	699 (6.9)	
Sigmoid Colon	3,340 (32.7)	3,340 (32.7)	
Histology			0.913
Adenocarcinoma	9,431 (92.4)	9,445 (92.6)	
Mucinous adenocarcinoma	748 (7.3)	733 (7.2)	
Signet ring cell carcinoma	24 (0.2)	25 (0.2)	
T stage			0.850
T3	8,668 (85.0)	8,641 (84.7)	
T4a	786 (7.7)	806 (7.9)	
T4b	749 (7.3)	756 (7.4)	
Age at diagnosis (years)			0.998
≤49	1,048 (10.3)	1,045 (10.3)	
>49–64	3,522 (34.5)	3,516 (34.5)	
>64–79	5,018 (49.2)	5,014 (49.1)	
>79	615 (6.0)	619 (6.1)	
Tumor size (cm)			0.999
≤2	352 (3.4)	355 (3.5)	
>2–4	3,358 (32.9)	3,351 (32.8)	
>4–6	3,594 (35.2)	3,596 (35.2)	
>6	2,899 (28.4)	2,901 (28.4)	
Tumor grade			0.952
Grade I	560 (5.5)	565 (5.5)	
Grade II	7,787 (76.3)	7,798 (76.4)	
Grade III/IV	1,856 (18.2)	1,840 (18.0)	

CT, chemotherapy; PSM, propensity score matching.

**Figure 4:**
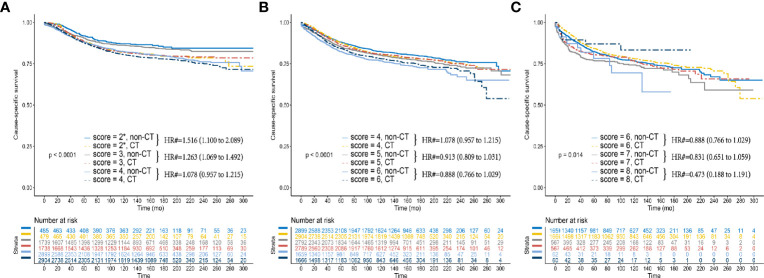
Kaplan–Meier CSS curves between the CT and non-CT groups after PSM in **(A)** *P* scores 2, 3, and 4 **(B)** *P* scores 4, 5, and 6 **(B)** *P* scores 6, 7, and 8. (2*) Including *P* scores 0, 1, and 2. (#) Multivariate analysis adjusted by the year of diagnosis, race, gender, tumor location, histology, T stage (including T3, T4a, or T4b), age at diagnosis, tumor size, and tumor grade.

**Figure 3B** showed that the overall survival (OS) benefit improved gradually when the score increased without exception, and the decline of 10-year OS rate among the non-CT group was much faster than among the CT group, which further validated the above findings. In addition, the Kaplan–Meier CSS curves of different *P* scores were also plotted, which also demonstrated the increased survival benefit offered by adjuvant chemotherapy as *P* score increased (P < 0.05, **Figures 4A–C**).

### Survival Benefit of Adjuvant Chemotherapy According to the *P* score Between T3 and T4 Groups

**Figure 5:**
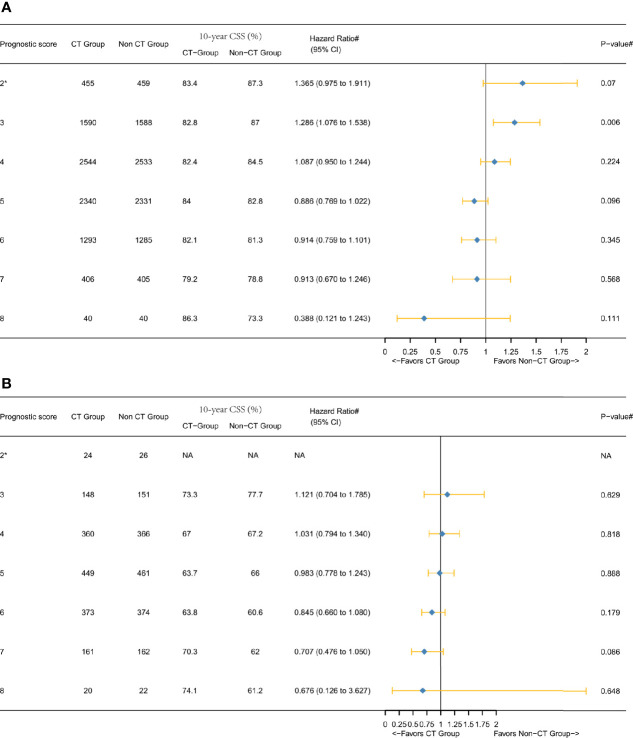
Hazard ratios comparing the CSS between the CT and non-CT groups according to *P* score in the subgroups after PSM comparing **(A)** T3 subgroup and **(B)** T4 subgroup. (2*) Including *P* scores 0, 1, and 2. (#) Multivariate analysis adjusted by the year of diagnosis, race, gender, tumor location, histology, T stage (T4 subgroup analysis, including T4a or T4b), age at diagnosis, tumor size, and tumor grade. (NA) Not applicable.

Next, we furtherly conducted the subgroup analyses and **Figure 5** showed the results of multivariate Cox and Kaplan-Meier analyses of CSS among both T3 and T4 subgroups. In the T3 subgroup analysis, it was also found that the 10-year CSS rate was higher in score 8 (86.3%) than that in score 7 (79.2%) among the CT group (**Figure 3A**). In the T4 subgroup analysis, a notable phenomenon we called “survival inversion” was that 10-year CSS rate increased gradually instead of decreasing when the score increased from 6 to 8 (**Figure 3B**). Thus, the “survival inversion” effect as *P* scores increased was even more pronounced among the T4 subgroup than among T3 subgroup. And the magnitude of CSS improvement offered by adjuvant CT was positively correlated with the *P* scores in both T3 and T4 subgroups. More importantly, more patients in the T4 subgroup favored adjuvant CT than in the T3 subgroup.

## Discussion

The majority of the randomized controlled trials (RCTs) regarding adjuvant CT in stage II colon cancer mixed the study population together with stage II and stage III diseases; only one RCT had focused on adjuvant CT in stage II colon cancer; however, the study found that high-risk stage II colon cancer did not benefit from 1-year adjuvant treatment with oral tegafur-uracil (UFT) (11, 17, 18). Although lack of sufficient evidence, ASCO and ESMO recommended the adjuvant chemotherapy in stage II colon cancer with the so-called high-risk prognostic factors (6, 7).

Furthermore, a unified definition of “high-risk” was absent as many countries had their different rules for risk assessment (19–22). In addition, ASCO (including inadequately sampled nodes, T4 lesions, perforation, or poorly differentiated histology) and ESMO (including lymph nodes sampling <12; poorly differentiated tumor; vascular or lymphatic or perineural invasion; tumor presentation with obstruction or tumor perforation and pT4 stage) clinical guidelines were different (6, 7). On the other hand, we could not quantify the necessity of adjuvant CT among stage II disease with high-risk factors considering they were only several independent prognostic factors (8).

Many clinical studies suspected the survival improvement of adjuvant CT in stage II colon cancer with high-risk factors (8, 11–14). In 2011, a large retrospective population-based clinical study found that adjuvant CT did not improve the overall survival substantially in stage II colon cancer either with or without high-risk prognostic features (including obstruction, perforation, emergent admission, T4-stage, resection of <12 lymph nodes, and poor histology) (14). A wide clinical application of adjuvant CT in stage II colon cancer with high-risk factors in spite of the uncertainty of survival benefit which could result in the overtreatment or undertreatment in stage II colon cancer. In addition, a significant patient morbidity could result from toxicity and side effects caused by adjuvant chemotherapy of overtreatment (23).

In this large population-based and PSM study, the current findings indicated that stage II colon cancer with higher *P* score (older patients, higher tumor grade, and larger tumor size) might be associated with improved CSS benefit of adjuvant CT. This phenomenon is of great clinical significance as we can predict the survival benefit of adjuvant CT well in stage II colon cancer using a simple *P* score. Considering that the *P* score is based on the tumor size, age, and tumor grade, which could be acquired before the operation, we could predict the survival benefit of adjuvant CT well among stage II disease preoperatively. Also, this study showed a successful validation of OS benefit improvement with increasing *P* scores (**Figure 3B**).

Our previous study demonstrated incremental mortality risk with increasing *P* scores among stage II disease (15). And it was also observed in the non-CT group that could validate our previous finding, yet we also noted that the phenomenon was slightly different among the CT group: the highest *P* score did not generate the lowest CSS rate either in T3 or T4 subgroup (**Figures 3** **–** **5** and **Figure S1**). The different phenomenon was more distinct in T4 subgroup analysis of CT group as 10-year CSS rate increased gradually instead of decreasing when the score increased from 6 to 8 (**Figure 3B**). This phenomenon was termed as “survival inversion” that could be attributed to the improvement in the survival benefit offered by adjuvant CT, contrary to decreased survival when *P* scores increased in the non-CT group. Moreover, the “survival inversion” was evident T4 subgroup than in the T3 subgroup.

In 2014, Aalok et al. (24) reported that the survival benefit of adjuvant CT was primarily observed in the T4 disease, thereby suspected the effect of adjuvant CT in stage II colon cancer with non-T4 high-risk factors. The study indicated that the several high-risk factors were not equivalent. Moreover, Matsuda et al. (11) reported that lymphatic invasion and poorly differentiated histology did not have any impact on the relapse-free survival of stage II colon cancer though they were listed as “high-risk” factors. Then, two studies from the United States and Netherlands proved that T4 had the maximum survival benefit with adjuvant therapy (8, 13). The results of the present study also showed that patients with lower *P* scores in the T4 subgroup were more likely to favor adjuvant CT as compared to the T3 subgroup in the prognostic scoring system, which was consistent with the previous studies, and it could lead to the speculation that *P* score might replace the role of high-risk factors in stage II disease.

The main strength of our study was the investigation of the survival benefit offered by adjuvant CT in stage II colon cancer according to the individualized patient risk factors. Based on the results of this large population-based and strictly PSM study with a long median follow-up time of about 10 years in the censored subjects, it was possible to guide the individual treatment decisions based on different *P* scores that could predict the survival benefit of adjuvant CT well in stage II disease. The “survival inversion” that reflected the association between tumor biology and clinical treatment also necessitates further exploration.

Nevertheless, the present study has some limitations. First, new biomarkers, such as RAS mutation, microsatellite instability, and carcinoembryonic antigen (CEA) level were studied intensively (18, 25–27). *P* score did not take other prognostic factors into account, indicating that *P* score requires further improvement. However, as a simple and convenient prognostic scoring system, *P* score could be obtained and calculated easily. Second, due to the limitation of SEER database, we cannot differentiate the chemotherapy regimens of CT, preoperative CT, postoperative CT, and the CT regimens. Considering it was not the standard therapy plan to treat stage II disease with preoperative CT, we can stratify the variable of “patient had chemotherapy” as “adjuvant CT.” Third, the statistical power was limited because some individual subgroups, such as score 0 and 8, were small after stratifying in spite of a large initial study population from SEER database. And survival difference was not statistically significant in some P score subgroups, which was consistent with previous large population-based study (28). Forth, some factors, such as clinical presentation with obstruction or perforation and disease-free survival data, were not available in the SEER database, were therefore not included in the present study. Finally, because a very large sample size was required to validate the clinical value of *P* score, we cannot conduct relevant analyses in our center, and the value of *P* score needed to be confirmed in large multi-center studies, especially in prospective cohorts.

## Conclusions

Here, based on the results of this large population-based and strictly PSM study with a long median follow-up time of about 10 years, our study demonstrated the improved survival benefit offered by adjuvant CT as *P* score increased, which can be used to guide the individual treatment decisions and predict the efficacy of adjuvant CT well in patients diagnosed with stage II colon cancer. In addition, *P* score was also easily obtained and calculated, meaning it could be of great clinical significance in therapy decisions in stage II colon disease. However, future studies focused on *P* score with prospective design were also essential.

## Data Availability Statement

Publicly available datasets were analyzed in this study. These data can be found here: The Surveillance, Epidemiology, and End Results (SEER) Program (https://seer.cancer.gov/).

## Author Contributions

QLi and XL conceptualized and designed the study. QLiu and ZS conducted the analyses of the study. QLiu, ZS, and DL interpreted the data. QLiu drafted the manuscript. QLiu, ZS, and DL revised the manuscript. All authors contributed to the article and approved the submitted version.

## Funding

This research was supported by the National Science Foundation of China (Nos. 81772599, 82002489, 81972260, and 81702353) and Shanghai Municipal Natural Science Foundation (17ZR1406400). The funders had no role in the study design, data collection and analysis, decision to publish, or preparation of the manuscript.

## Conflict of Interest

The authors declare that the research was conducted in the absence of any commercial or financial relationships that could be construed as a potential conflict of interest.

## Supplementary Material

Supplementary Figure 1 Hazard ratio comparing the cause-specific survival (CSS) between chemotherapy (CT) and non-CT groups according to the *P* score in the overall cohort before propensity score matching (PSM). (2*) Including *P* scores 0, 1, and 2. (#) Multivariate analysis adjusted by the year of diagnosis, race, gender, tumor location, histology, T stage (including T3, T4a, or T4b), age at diagnosis, tumor size, and tumor grade.
