# Supplementary figures and images for: Associations of P Score With Real-World Survival Improvement Offered by Adjuvant Chemotherapy in Stage II Colon Cancer: A Large Population-Based Longitudinal Cohort Study
Source: Front Oncol. 2021 Feb 24;11:574772. doi: 10.3389/fonc.2021.574772 (PMC7945037; doi:10.3389/fonc.2021.574772)

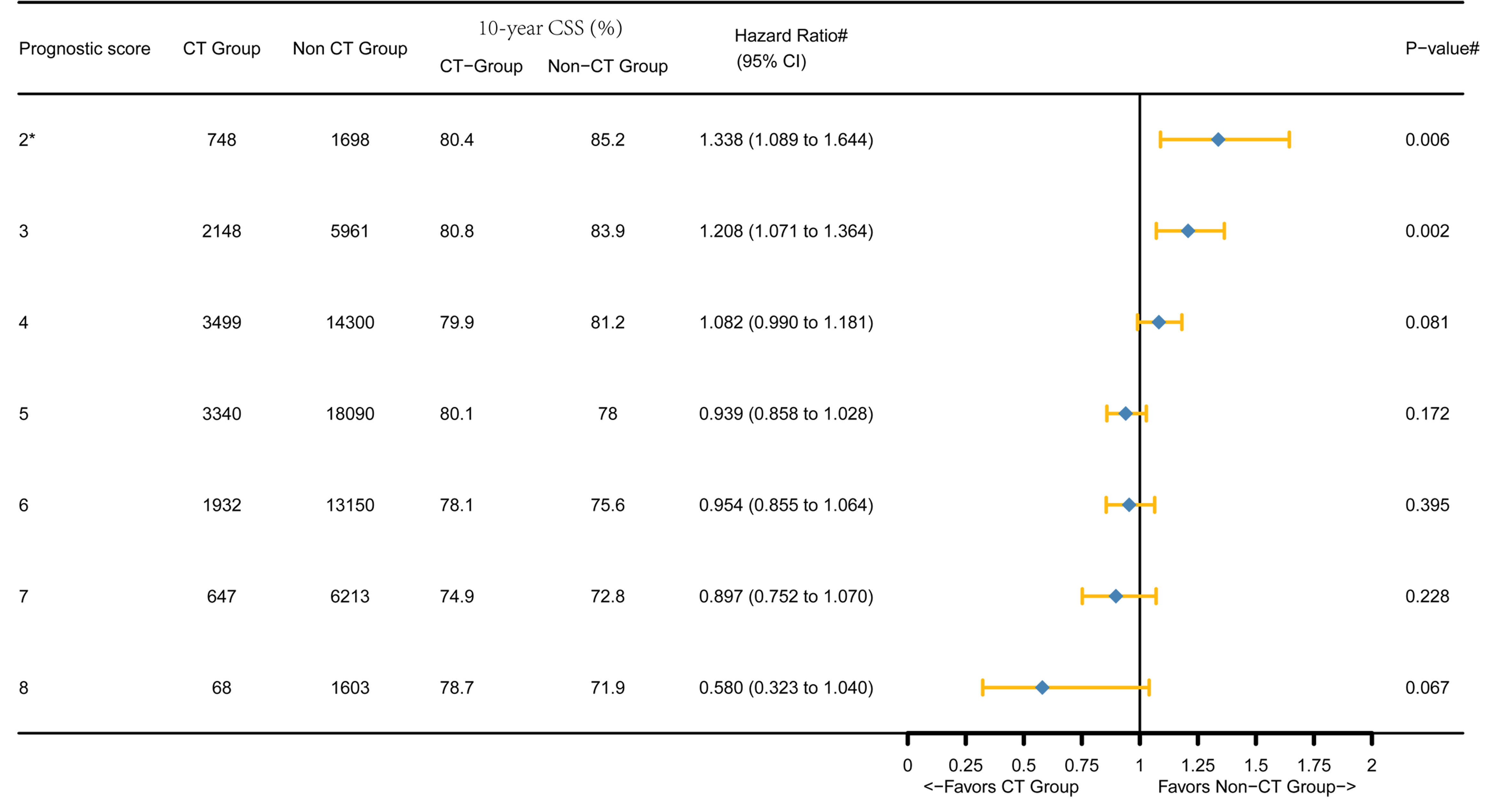

Supplement: Supplementary Figure 1 — Hazard ratio comparing the cause-specific survival (CSS) between chemotherapy (CT) and non-CT groups according to the P score in the overall cohort before propensity score matching (PSM). (2*) Including P scores 0, 1, and 2. (#) Multivariate analysis adjusted by the year of diagnosis, race, gender, tumor location, histology, T stage (including T3, T4a, or T4b), age at diagnosis, tumor size, and tumor grade. [file Image_1.jpeg]
